# Cancer and Pregnancy in the Post-Roe v. Wade Era: A Comprehensive Review

**DOI:** 10.3390/curroncol30110684

**Published:** 2023-10-25

**Authors:** Ganguly Arup, Narmala Shravan

**Affiliations:** 1 Department of Internal Medicine, University of Connecticut Health Center, Farmington, CT 06030, USA; 2 Department of Hematology and Oncology, DHR Health Oncology Institute, Edinburg, TX 78539, USA; s.narmala@dhr-rgv.com

**Keywords:** pregnancy, abortion, cancer, immunotherapy, Roe v. Wade, chemotherapy

## Abstract

Cancer during pregnancy, affecting 1 in 1000 pregnancies, is rising in incidence due to delayed childbearing and improved detection. Common types include breast cancer, melanoma and cervical cancer and Hodgkin’s Lymphoma. There are several physiological changes that occur during pregnancy that make its management a challenge to clinicians. Managing it requires multidisciplinary approaches and cautious test interpretation due to overlapping symptoms. To minimize fetal radiation exposure, non-ionizing imaging is preferred, and the interpretation of tumor markers is challenging due to inflammation and pregnancy effects. In terms of treatment, chemotherapy is avoided in the first trimester but may be considered later. Immunotherapy’s safety is under investigation, and surgery depends on gestational age and cancer type. Ethical and legal concerns are growing, especially with changes in U.S. abortion laws. Access to abortion for medical reasons is vital for pregnant cancer patients needing urgent treatment. Maternal outcomes may depend on the type of cancer as well as chemotherapy received but, in general, they are similar to the non-pregnant population. Fetal outcomes are usually the same as the general population with treatment exposure from the second trimester onwards. Fertility preservation may be an important component of the treatment discussion depending on the patient’s wishes, age and type of treatment. This article addresses the complicated nature of a diagnosis of cancer in pregnancy, touching upon the known medical literature as well as the ethical–legal implications of such a diagnosis, whose importance has increased in the light of recent judicial developments.

## 1. Introduction

Cancer during pregnancy poses unique challenges to maternal and fetal well-being. Pregnancy-Associated Cancers (PAC) are defined as cancers diagnosed in the pregnancy and/or the post-partum period. Published data indicate that such cancers occur in approximately 1 in 1000 pregnancies [1]. The most common malignancies during pregnancy include breast cancer, melanoma, cervical cancer, lymphomas, and leukemias. The increasing prevalence of cancer during pregnancy, influenced by delayed childbearing and improved detection, emphasizes the need for optimized care. Obtaining an accurate diagnosis while minimizing fetal harm is a delicate balance, involving multidisciplinary approaches and the cautious interpretation of tests [2].

Following the emergence of targeted therapy and newer treatments, as well as the establishment of clear guidelines to allow for a more standardized approach, can ensure the best outcomes for both mother and child.

Ethical considerations involve respecting maternal autonomy and optimizing outcomes through shared decision-making and open communication. This review focuses on enhancing cancer management in pregnancy, covering the diagnosis, treatment considerations, surveillance, and ethical complexities, and discussing the implications of the overturning of the Roe v. Wade ruling on access to cancer treatment for pregnant individuals.

## 2. Epidemiology and Risk Factors

The prevalence of cancer during pregnancy is increasing, and these patients are at increased risk for poor outcomes. According to a recent nationwide study from the National Inpatient Sample, the prevalence rate of pregnancy with cancer was 69.3 per 100,000 deliveries and increased from 64.5 to 73.4 between 2016 and 2019 (relative increase, 13.8%; *p* < 0.001) [3]. Cancer was associated with a range of factors, including patient characteristics such as advanced age, a more recent year of delivery, White ethnicity, obesity, smoking, preexisting hypertension, and exposure to chemotherapy. Additionally, pregnancy characteristics like early preterm delivery and cesarean delivery were also linked to cancer. Table 1 shows the incidence of various types of cancer commonly associated with pregnancy in the population of the United States [4,5,6,7,8,9,10,11,12,13].

## 3. Diagnostic Challenges

### 3.1. Impact of Pregnancy on Cancer Diagnosis

Pregnancy involves a change in the physiology of the body and has a high metabolic demand, which is amplified by the detection of a cancer. The physiological changes that occur during pregnancy may contribute to the masking of cancer symptoms [14]. There are several overlapping signs and symptoms of cancer and pregnancy, including nausea and vomiting, appetite changes, constipation, abdominal discomfort, anemia, a palpable breast mass/increased volume and consistency of breast tissue, hyperpigmentation, and fatigue [15,16]

### 3.2. Considerations for Imaging Modalities and Radiation Exposure

It is important to select appropriate imaging studies that minimize stress and avoid the unwanted termination of pregnancy. Non-ionizing modalities such as ultrasound (US) and Magnetic Resonance Imaging (MRI) are considered first-line options. When Computed Tomography (CT) scans are used, radiation dose and subsequent fetal dose should generally not exceed 0.10 Gray (Gy) [17]. CT of the head, neck, thorax, and extremities is generally considered safe. PET/CT and PET/MRI are used for staging and assessing treatment response [18]. Since PET/CT involves both radioisotopes and CT, it results in a higher total radiation dose compared to CT alone. Therefore, the use of PET/CT should be carefully considered in pregnancy. On the other hand, a PET/MRI is another viable modality, with early studies showing some benefit [19,20]. A fetal dose of 0.1 Gy carries a small individual risk of radiation-induced cancer, but more than 99% of exposed fetuses will not develop childhood cancer or leukemia [18].

Several radioisotopes are considered unsafe in pregnancy. Some of them may cross the placenta and remain in the fetus for several days and can cause organ damage [18]. In general, therapeutic radiopharmaceuticals should be avoided during pregnancy, except in cases where the mother’s life is at risk. In such situations, careful assessment of the fetal dose and gestational age (GA) may be necessary, and the possibility of the termination of pregnancy should be considered.

## 4. Challenges in Interpreting Tumor Markers during Pregnancy

Specific tumor markers are produced not only by tumor cells but also in response to inflammation. Increased levels of tumor markers are also associated with pregnancy. Variations in levels are even more prominent in pregnancies complicated by obstetric issues [21]. For example, alfa-fetoprotein (AFP) is a marker for hepatocellular carcinoma and is elevated in the maternal circulation by fetal production, and this level may be up to 13 times higher in pregnancy complications such as preeclampsia [22]. Similarly, levels of CA-125 may be elevated in pregnancy, especially in the first trimester [23]. During breast cancer and pregnancy, carbohydrate antigen 15-3 (CA 15-3) levels significantly increase, especially in the third trimester, showing a 3.3–20.0% increase above cut-off levels [21,24]. There are, however, traditional tumor markers that seem to be unaffected in pregnancy, including lactate dehydrogenase (LDH), Inhibin-B, anti-Mullerian hormone (AMH), carcino-embryonic antigen (CEA), CA-19-9, and human epididymis protein 4(HE4) [25,26,27].

The utilization of single-day protocols for sentinel node procedures during pregnancy is generally regarded as a safe and well-tolerated approach [28]. When administered at low doses that do not exceed a fetal exposure of 5 mGy, radiopharmaceuticals can be effectively employed for these procedures, offering a viable and safe method that ensures both maternal and fetal well-being [29].

## 5. Treatment Considerations

### 5.1. Multidisciplinary Approach in Managing Cancer during Pregnancy

The diagnosis of cancer during pregnancy is an extremely sensitive situation, requiring delicate and specialized care by a multidisciplinary team. It is also important that such cases are managed at a higher-level care center, with the access to resources and specialists necessary to address all aspects of treatment [2]. It is important for a maternal fetal medicine specialist and the relevant oncology specialists, along with the primary care provider and an appropriate mental health professional, if required, to be involved. This constitutes high-value care covering all aspects of the mother’s and fetus’ well-being.

A recent article by Silverstein et al., the authors suggest counseling patients about pregnancy termination when appropriate for the kind of cancer and the gestational age at diagnosis, especially in cases of an aggressive or advanced stage of cancer being found early in pregnancy [30]. However, according to Wolters et al., no studies have shown an improved maternal prognosis, as very few case control studies exist on this topic [28]. They recommend against the termination of pregnancy solely for the purpose of improving maternal outcomes.

### 5.2. Chemotherapy

Chemotherapy is generally avoided during the first trimester as this may lead to significant morbidity, especially congenital malformations [31,32,33]. A recent cohort study found that the major congenital malformation rate among offspring was as high as 21.7% (95% CI 7.5–43.7%) when associated with maternal exposure to chemotherapy prior to 12 weeks, compared with 3% in women who received chemotherapy after 12 weeks (95% CI, 3.13–27.30) [34]. The nature and mechanisms by which chemotherapy induces these malformations is not clearly understood and involves a variety of factors, including genetic susceptibility, the timing of exposure to chemotherapy and the type of drug used [34] Defects in the eyes, genitalia, and central nervous system become obvious after birth and develop throughout infancy and childhood [34,35].

As previously mentioned, in cases of advanced or aggressive cancers, counseling patients regarding the termination of pregnancy is appropriate. Chemotherapy is generally avoided during the first trimester of pregnancy to avoid interference with organogenesis. There is a strict association between the initiation of chemotherapy and congenital malformations in the first trimester, namely defects in the heart, limbs, neural tube, palate, eyes and ears [36,37]. The risk of malformations is reduced after this period and the incidence is similar to the general population, without cancer and not receiving chemotherapy [38]. Drug passage through the placenta depends on factors like protein binding, lipid solubility, and ionization constant. Fetal exposure to drugs is influenced by maternal pharmacokinetics, such as the volume of distribution, placental metabolism and excretion rate, pH difference between maternal and fetal fluids, and hemodynamic changes during pregnancy. Although several chemotherapeutic agents have a low molecular weight, are lipid-soluble and are non-ionized, which are all factors that favor passive diffusion, concentrations of these drugs are found to be significantly lower than in the maternal circulation. The administration of most cytotoxic drugs is considered relatively safe after the first trimester [38,39]

After reaching the 35-week gestational mark, the medical consensus typically leans toward avoiding chemotherapy. This precautionary measure is taken to allow for a substantial duration for the maternal and fetal bone marrow to recuperate adequately following the conclusion of the last chemotherapy cycle, thereby optimizing the health and well-being of both the expectant mother and the developing fetus in the lead-up to delivery [28]. Breast feeding should be avoided in patients receiving chemotherapy [40].

### 5.3. Immunotherapy and Targeted Agents

The advent of immunotherapy in treating cancer in non-pregnant patients over the years has fostered great research, in the form of clinical trials, but studies on the use of these agents in pregnancy is limited.

During pregnancy, the maternal immune system faces the task of tolerating the fetus while also protecting against infections and toxins. This process happens in three phases: first, a pro-inflammatory state supports implantation and placenta formation; second, an anti-inflammatory environment develops in the uterus during the second and third trimesters; finally, a second pro-inflammatory stage triggers uterine contractions, fetal delivery, and placental expulsion during parturition [41]. In the realm of immunotherapy, immune checkpoint inhibitors (ICIs) stand out as the prevailing class of therapeutic agents. This category encompasses inhibitors targeting programmed cell death protein 1 (PD-1), programmed cell death ligand 1 (PD-L1), and cytotoxic T-lymphocyte antigen 4 (CTLA-4), which are widely recognized and employed in clinical practice. Among the various other agents utilized in this context, there are several noteworthy ones, such as certain cytokines, primarily interferon alpha (IFNα) and interleukin-2 (IL-2). Additionally, the use of vaccines, such as Bacillus Calmette–Guérin (BCG), and genetically engineered type 1 herpes simplex viruses, along with specific T-cell engagers, have also been explored as potential therapeutic approaches [42].

Current guidelines advise against ICI use in patients of childbearing age (unless using effective contraception) during and for at least 5 months after the last dose of ICI treatment [43]. A recent study sought to characterize the safety profile of ICIs in pregnancy using the World Health Organization’s (WHO’s) spontaneous reporting system by way of a disproportionality study. In this study, a total of 56 patients reported pregnancy-related outcomes. The most common reported maternal outcomes were diarrhea (5.4%), nausea, fatigue, abdominal pain, pruritis and chest pain (3.6%). The most common fetal outcomes included spontaneous abortions (21.4%) and prematurity (32.1%). Uncomplicated deliveries occurred in 7.1% of cases [44]. With regards to other immunotherapeutic agents, there are limited data on the safety profile in terms of risks to the neonate.

An analysis of clinical trials by Mittra et al. showed that, in a 10-year period from 2011 to 2020, seven patients who had a diagnosis of cancer during pregnancy and who decided to take their pregnancy to term decided to continue treatment with immunotherapeutic agents. All pregnancies resulted in vaginal births of apparently normal infants [45].

### 5.4. Surgical Interventions and Potential Effects on the Fetus

Surgery is generally not a contraindication regardless of gestational age. For gynecological malignancies, it is preferably performed in the early second trimester when the risk of miscarriage is lower and uterine size allows access [14]. For oncologic procedures, the left lateral tilt position is advised due to the operating time and general anesthesia use, while the right lateral tilt can be considered if it improves exposure [46]. The feasibility of laparoscopy during pregnancy varies based on gestational age, surgeon’s experience, procedure type, and the organs involved. A study comparing laparotomy to laparoscopy in pregnant women revealed that laparoscopy resulted in fewer fetal adverse effects, shorter operative times, and reduced hospital stays [47].

## 6. Ethical and Legal Perspectives

In a historic ruling on 22 January 1973, the United States Supreme Court ruled in favor of a woman’s right to have an abortion. This meant that the decision on whether or not to have an abortion fell within the realm of individual authority and not government authority. This established the Fourteenth Amendment’s “liberty” guarantee, safeguarding personal privacy, encompassing the right to abortion before fetal viability. Since then, the Supreme Court has consistently upheld this constitutional protection for abortion as a fundamental aspect of liberty, interconnected with the ability to make personal choices concerning family, relationships, and bodily autonomy. However, on 24 June 2022, this landmark ruling was overturned, ending national protection to this constitutional right [48]. There were approximately 3.6 million live births in the United States in 2022, and a total of 21 states have now either completely banned or imposed restrictions on abortion at the time of writing this article. This means that approximately 40% of live births occurred in states where abortion is restricted or banned. At least 1500 individuals will develop pregnancy-associated cancer in these states. Between 135 and 420 individuals with pregnancy-associated cancer will receive suboptimal care and may face mortality because an abortion needed to start therapy may not be protected by law [49].

The embryo or fetus’s age significantly impacts access to pregnancy termination as governed by evolving state laws. Medical viability begins around 24 weeks, with a high risk of severe prematurity until 28 weeks, linked to neonatal issues. Even though late-preterm complications are rare (34–36 weeks), experts suggest supporting the fetus until 37 weeks for better outcomes [30,50]. When a viable yet preterm fetus conflicts with the safety of the mother’s therapy, balancing the risks of early delivery against delayed oncologic treatment becomes crucial. Complex medical choices are frequently swayed by religious, cultural, and personal beliefs, alongside factors like family dynamics, community influence, and trust in healthcare providers. Notably, some religions and cultures oppose ending pregnancies, even if it endangers the mother’s life. In instances where a mother receives a terminal diagnosis, the idea of raising a child without her and the financial burden can be inconceivable. Additionally, a first-time mother’s decisions about her survival versus the fetus may contrast with those of a mother already caring for dependents.

Pregnancy termination constraints will mainly impact cases where urgent oncologic therapy is required, yet contradicted due to pregnancy, and the fetus is not viable. Decisions about emergency terminations or when life is at risk hinge on specific state regulations. Acts like the Emergency Medical Treatment & Labor Act (EMTALA) guarantee emergency care to all patients, irrespective of their financial status [51,52]. Recent federal guidance reaffirmed that abortion is allowed in cases of medical emergencies, such as ectopic pregnancy or severe preeclampsia, regardless of state regulations [53]. While cancer can be life-threatening, it is uncertain if it qualifies as an emergency warranting immediate treatment under these provisions.

Lawmakers refer to the “constitutional right to interstate travel” as an option to seek abortion in another state, but ongoing debates continue [54]. This safeguard may only benefit those with the means to travel, disproportionately impacting disadvantaged patients. Oncologists practicing in states with abortion restrictions will face challenges in advising terminations based on medical reasons.

## 7. Maternal and Fetal Outcomes

### 7.1. Maternal Prognosis and Long-Term Effects of Cancer Treatment

Maternal outcomes may be cancer-specific. In general, there is not much difference in cause-specific deaths between pregnant or lactating women compared to non-pregnant women with cancer, as seen in a cohort study conducted by Stensheim et al. [55]. In this study of 42,511 women who were diagnosed with cancer during pregnancy between 1967 and 2002, outcomes were monitored over a median period of 9 years. They were grouped as non-pregnant, pregnant or lactating at diagnosis. Across all sites, there were no significant differences in cause-specific death rates between groups. Within the pregnant group, the HR stood at 1.03, accompanied by a 95% confidence interval (CI) ranging from 0.86 to 1.22. Conversely, the lactating group displayed a marginally lower HR of 1.02, with a corresponding 95% CI of from 0.86 to 1.22. A specific subset of patients diagnosed with breast cancer demonstrated a substantially higher HR of 1.95. This increased risk of cause-specific death was statistically significant, as evidenced by the 95% CI ranging from 1.36 to 2.78. Similarly, for patients diagnosed with ovarian cancer during lactation, the HR climbed to 2.23, again with a notable 95% CI, this time spanning from 1.05 to 4.73. These observations underscore the critical impact of timing, specifically lactation, on the prognosis for patients facing these particular cancer types, warranting focused attention and further investigation into the underlying factors contributing to this elevated risk. A slight increase in this risk was observed for malignant melanoma diagnosed during pregnancy. Interestingly, women with post-cancer pregnancies experienced a significant decrease in the risk of cause-specific death across all cancer types [55].

Within the realm of breast cancer research, a meta-analysis, led by Hartman et al. [56], assessed overall survival (OS) and disease-free survival (DFS) among a diverse cohort. This cohort consisted of patients diagnosed with breast cancer either during their pregnancy or up to five years after childbirth. The subanalysis of these data brought to light co insights into the varied survival outcomes within these subgroups. The subgroup encompassing patients diagnosed with breast cancer during pregnancy or postpartum (PABC) demonstrated a distinct, less favorable trend in overall survival (OS). The hazard ratio (HR) for this particular subgroup was estimated at 1.46, as substantiated by the 95% confidence interval (CI) ranging from 1.17 to 1.82. This marked an increased risk of adverse outcomes. Furthermore, the investigation singled out patients diagnosed with breast cancer during pregnancy alone, with the results indicating a similarly heightened risk of compromised OS. The HR in this context was found to be 1.47, and the 95% CI spanned from 1.04 to 2.08, when compared to non-pregnant women of the same age. Similar results were seen in DFS among patients with pregnancy-associated breast cancer (HR 1.51; 95% CI 1.22–1.88). Pregnant women diagnosed with malignant melanoma exhibited a heightened risk of cause-specific death (HR 1.52). However, cervical, thyroid, and ovarian cancer, as well as lymphoma or leukemia, did not exhibit significantly altered cause-specific death risks (HR 1.23, HR 0.89, HR 1.15, HR 4.58, and HR 0.46, respectively) [56].

In another study, patients diagnosed with pregnancy during cancer and in the first-year post-partum were found to be in more advanced stages of the disease compared to non-pregnant patients. Additionally, for all cancer types, patients with pregnancy-associated cancer had a similar overall survival with a hazard ratio (HR) of 1.07 (95% CI 0.80–1.41) for the pregnancy group and HR 1.02 (95% CI 0.88–1.18) for the postpartum group [57] In general, maternal outcomes seem to depend on the type of cancer and the stage at which they are diagnosed but, when combined, outcomes appear to be similar to non-pregnant patients.

### 7.2. Fetal Outcomes

A multicenter case-control study conducted by Amant et al. [58] studied fetal outcomes in mothers who received chemotherapy, radiation, and had surgery during pregnancy.

Throughout pregnancy, chemotherapy, either as a sole treatment or in combination with other therapies, was administered to 74% of children, while 8.5% received radiotherapy, either as a standalone treatment or in combination. A further 10.1% underwent surgery as their exclusive therapeutic intervention, 1.6% were treated with alternative drug therapies, and 10.9% received no treatment. It is noteworthy that, in the prenatal-exposure group, 22.0% of infants exhibited birth weights below the 10th percentile, in contrast to 15.2% in the control group, although this difference was not statistically significant (*p* = 0.16). Furthermore, cognitive development did not display any notable variation between the two groups. Moreover, a cardiologic evaluation conducted on 47 children at 36 months of age revealed that cardiac findings remained within the normal range [58].

In another multicenter cohort study examining outcomes at 6 years post-partum with a sample of 132 children, statistically significant differences were found in mean verbal Intelligence Quotient (IQ) and visuospatial long-term memory. Additionally, a significant difference in diastolic blood pressure was found, with higher values in the chemotherapy-exposed group. Additionally, ototoxicity was found in 3 cases of exposure to cisplatin. There were, however, no differences in overall IQ [59].

Cardonick et al. reported that the majority of children exposed to chemotherapy did not show any significant complications [35]. A study led by Murthy et al. indicates that administering 5-fluorouracil, doxorubicin, and cyclophosphamide to pregnant women with breast cancer during the second and third trimesters is a safe therapeutic option. This treatment approach does not appear to raise significant concerns regarding severe complications or immediate health risks for offspring exposed to chemotherapy in utero [60]. Another study found that the incidence of birth defects of infants followed up to 7 years was the same as the CDC-reported national average of 3% [61].

It is necessary to monitor newborns very closely to observe for side effects and the assessment must be made on a case-by-case basis depending on the type of cancer and type of treatment. Wolters et al. [28] recommend baseline screening with a neonatal complete blood count (CBC), especially if the last chemotherapy was within three weeks of delivery. Screening for cardiac abnormalities should be carried out in instances of anthracycline exposure with an echocardiogram during the neonatal period. Children who have been exposed to platinum-based chemotherapy should undergo screening for auditory impairment using otoacoustic emissions until they reach the age of 5, after which regular audiometry tests should be conducted at subsequent stages of development. Additionally, if placental metastases are suspected, a liver function test and abdominal ultrasound should be carried out. Close attention must be paid to developmental milestones in consultation with a pediatrician.

## 8. Preservation of Fertility

For many women, having children after being diagnosed with cancer is a key component of psychological well-being. Experiencing infertility on its own is linked to considerable psychological distress, resulting in depression rates twice as high as those found in the general population [62]. Additionally, this leads to a reduction in the quality of life concerning emotional well-being, relationships, and sexuality. A study by Schover et al. showed that 76% of cancer patients of child-bearing age wished to have children after recovering from cancer [63]. The risk of infertility with cancer treatment is mostly dependent on the patient’s age and the type of treatment received. Most fertility preservation techniques for women, such as embryo cryopreservation, oocyte preservation, ovarian tissue preservation, ovarian transposition and hormonal treatment, are not viable for use during pregnancy [64]. It is, therefore, prudent to offer these options following delivery, along with an evaluation of reproductive capacity.

## 9. Conclusions

A diagnosis of cancer during pregnancy adds to the many challenges that patients already face regarding the pregnancy itself. It is additionally challenging to all provider teams involved, and in the wake of the recent overturning of abortion laws it is important to have clarity and clear guidelines on how best to approach a situation that is already medically complex. Various tumor markers behave differently throughout the course of pregnancy. Chemotherapy and targeted therapies are generally avoided during the first trimester, but studies show that they are safe later during the pregnancy. Radiation is avoided throughout pregnancy. Maternal outcomes are generally favorable following delivery, but more studies are required to assess long-term fetal outcomes and side effects.

## Figures and Tables

**Table 1:** The incidence of various cancers in pregnancy per 100,000 pregnancies.

Type of Cancer	Incidence Per 100,000 Pregnancies
Breast cancer	33
Cervical intraepithelial neoplasia	200
Cervical cancer	1–120
Hodgkin’s lymphoma	16–100
Non-Hodgkin’s lymphoma	1–5
Leukemia	1
Ovarian cancer	3–6
Melanoma	39
Thyroid cancer	14
Colorectal cancer	2
Head and neck (non thyroid)	0.7
